# Immune Thrombocytopenia and JAK2V617F Positive Essential Thrombocythemia: Literature Review and Case Report

**DOI:** 10.1155/2017/3725089

**Published:** 2017-07-20

**Authors:** M. A. Sobas, T. Wróbel, K. Zduniak, M. Podolak-Dawidziak, J. Rybka, M. Biedroń, M. Sawicki, J. Dybko, K. Kuliczkowski

**Affiliations:** ^1^Department of Haematology, Blood Neoplasms and Bone Marrow Transplantation, Medical University of Wrocław, Wrocław, Poland; ^2^Department of Pathology, Medical University of Wrocław, Wrocław, Poland

## Abstract

We present the case where immune thrombocytopenia (ITP) and essential thrombocythemia (ET) sequentially appeared in the space of twenty-one years of follow-up. Impaired platelet production is present in both diseases, but clinical presentation and treatment are different. On the basis of this case history a possible role of autoimmunity as a predisposing factor to myeloproliferation has been discussed.

## 1. Introduction

Primary immune thrombocytopenia previously called idiopathic thrombocytopenic purpura or immune thrombocytopenic purpura (ITP) is characterized by autoimmune-mediated platelet destruction and suppression of megakaryocyte platelet production [1]. ITP is a diagnosis of exclusion. The definition of primary ITP by the International Working Group is platelet count less than 100 G/L without other reasons to explain thrombocytopenia [2].

Essential thrombocythemia (ET) is a clonal expansion of multipotential stem cells. Clonal thrombocytosis is a part of ET and is found also in 50% of patients with polycythemia vera and 35% of patients with chronic myeloid leukemia. Similarly to ITP, ET is also a diagnosis of exclusion once other thrombocytoses such as reactive thrombocytosis, myelodysplastic syndrome (MDS), and other myeloproliferative neoplasms (MPN) have been eliminated. JAK2 V617F mutation was found in 50% of ET patients, CALR in 25%, and MPL in few %, and only about 15% of ET patients do not harbor any of driver mutations (triple negative cases) [3, 4].

Some data shows that autoimmune disorders, including immune thrombocytopenia, may precede the development of myeloproliferative neoplasms [5–8]. Others did not find any associations between autoimmune disorders and MPN Ph(−) but proved one with myelodysplastic syndrome (MDS) and acute myeloid leukemia (AML) [6].

Here we present a case report of a patient with ITP who was diagnosed with ET, after 21 years of follow-up.

## 2. Case Report

**Figure 1:**
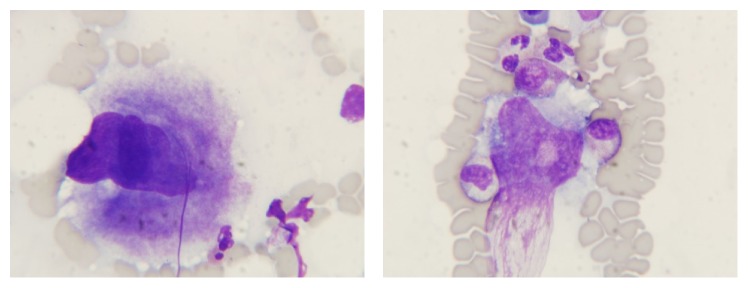
Bone marrow aspiration showed increased number of megakaryocytes, particularly those with hypolobulated nuclei. There was not any other abnormality in the bone marrow smear.

**Figure 2:**
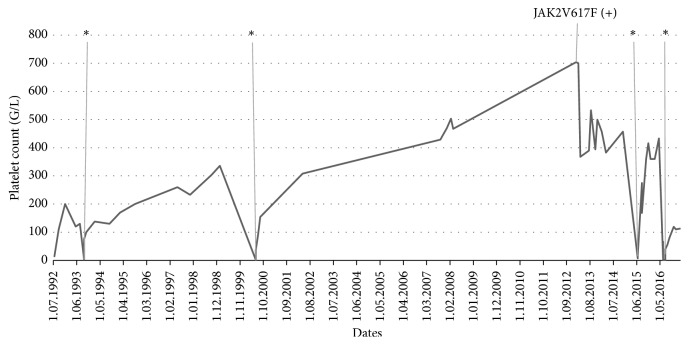
Kinetics of a platelet count during the follow-up of ITP-ET patient. ^*∗*^ITP relapse.

**Figure 3:**
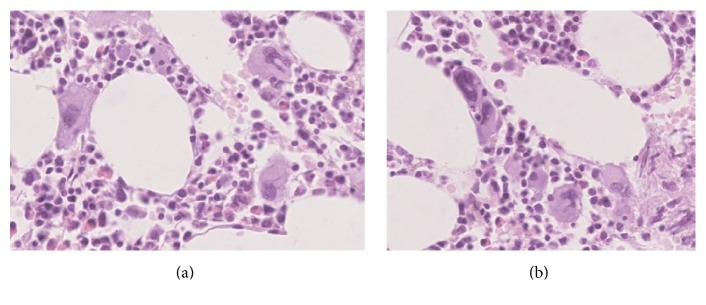
Trephine biopsy revealed normocellular bone marrow without fibrosis with normal differentiation of granulocytic and erythroid lineage. A marked increase in megakaryocytic density was present. Megakaryocytes presented with moderate pleomorphism, most cells showing nuclear hyposegmentation with normal nuclear to cytoplasmic ratio (a) and a few myeloproliferative hypersegmented and atypical forms. Moreover megakaryocytic lineage displayed focal aggregation (b).

The female patient was initially presented at the age of 45 in July 1992 with ecchymoses in the skin over limbs and arms and hemorrhagic bullae on oral mucous membrane. She admitted having menorrhagia and single ecchymoses a few weeks earlier. In 1987 she had an incidental mild asymptomatic thrombocytopenia but no other history of personal or family bleeding. There was no history of long term medication or any preceding infection. No constitutional symptoms, for example, weight loss, bone pain, or night sweats, were present. The lymph nodes, liver, and spleen were not enlarged. Gynecological examination was normal. Laboratory results shown isolated thrombocytopenia with platelet count 14 G/L as her hemoglobin level was 14 g/dL and white blood cell count was 8.6 G/L. There were no abnormalities in peripheral blood smear. Coagulation tests, C-protein level, and liver and kidney tests were normal. Tests for viral hepatitis B and hepatitis C, CMV, and HIV were all negative. Test for* Helicobacter pylori* was not performed. Antiplatelet antibodies examined by immunoenzymatic method (MAIPA) and ANA (antinuclear antibodies) were negative. Bone marrow aspirated biopsy (Figure 1) confirmed a peripheral thrombocytopenia. She was diagnosed as having immune thrombocytopenia (ITP). Prednisone in doses of 1 mg/kg of body weight was administered for six weeks, and platelet count rose above 100 G/L. No further medication was given and her platelet count was monitored closely. First relapse of ITP was observed in August 1993 and the second one in June 2000, and at both patient responded well to corticosteroids. Platelet counts remained stable till 2007 when they started to increase slowly and reached 704 G/L in January 2013 (Figure 2), while hemoglobin level was 15.4 g/dL and leukocytes were 5.85 G/L. The JAK2 V617F mutation was found (performed in DNA from peripheral blood leukocytes by PCR ARMS method). According to WHO 2008 diagnostic criteria, bone marrow biopsy was done, and it was compatible with ET (Figure 3). The patient had no previous incidents of thrombosis or hemorrhage; however as she was older than 65 years cytoreductive therapy with hydroxyurea (HU) has been introduced. The complete response was achieved, and platelet counts were stable until June 2015 when severe thrombocytopenia suddenly appeared (platelet count was 4.0 G/L). HU was stopped. A central thrombocytopenia was excluded on the basis of a picture of bone marrow aspirate. The presence of JAK2 V617F mutation was confirmed. The third relapse of ITP was diagnosed, and corticosteroids were restarted, but this time there was no response and IVIG in the dose 0.4 mg/kg/daily were given for five days with good effect. To maintain the response, azathioprine (AZT) was added (150 mg/day), and it was tapering slowly until stopped after four months, in November 2015. A month later patient had platelet count 466 G/L and HU was reintroduced in the dose 500 mg every second day. Platelet count remained stable till June 2016 when patient had a gastrointestinal infection and there was a severe thrombocytopenia with platelets 1.0 G/L. After exclusion of central thrombocytopenia in bone marrow the fourth ITP relapse was diagnosed. This time patient did not respond to corticosteroids or to IVIG. However, we decided to put her on low dose of prednisone (20 mg/day) plus AZT (150 mg/day). After one month on such a therapy platelet count increased up to 26.0 G/L, in August 2016 it was 47 G/L, and prednisone was slowly tapered off. Since September 2016 she was only on AZT, which was stopped in December 2016 when platelet count reached 110 G/L. The last control was performed in February 2017, and patient was in good condition with platelet count 113 G/L without any medication (Figure 2).

## 3. Discussion

We present a case where immune thrombocytopenic purpura and essential thrombocythemia were diagnosed in the space of eleven years. Interrelationship between autoimmunity and myeloproliferation is quite complex and not wholly understood.  Table 1 showed characteristic of our patient in comparison to two other cases in whom ITP was followed by ET [9, 10].

According to the literature, certain treatments for autoimmune conditions, such as azathioprine, could increase the risk of developing MDS or AML [11, 12]. There are also suggestions that chronic inflammation may drive clonal myeloproliferation [7, 8]. Autoimmune/inflammatory diseases may precede or develop during the course of MPNs [5, 7, 8]. Kristinsson et al., in a large population-based study performed on a cohort of 11,039 MPN patients versus 43,550 matched controls, found that a prior history of MPN development [odds ratio (OR) 1.2, 95% confidence interval (CI) 1.0–1.3, and *p* = 0.021], specifically ITP, Crohn's disease, rheumatic polymyalgia, giant cell arteritis, Reiter's syndrome, and aplastic anaemia, was associated with the risk of MPN [5]. In a study by Anderson et al. [6] conducted on an American population with 13.486 myeloid malignancy patients and 160.086 controls, association between any prior autoimmune disease and the risk of myelodysplastic syndrome (MDS) and acute myeloid leukemia (AML) was reported. This relation was not seen in MPN. Based on a potent anti-inflammatory effects of JAK2-inhibitors, Hasselbalch postulated that their early introduction in treatment of MPN patients, especially in case of patients with myelofibrosis, may change the course of the disease [7, 8]. The sequential occurrence of two different types of platelet disorder is rarely reported in the literature [9, 10]. Autoimmune disorders can act as possible predisposing factors for myeloproliferative neoplasms development [5–8].

It can only be speculated that autoimmunity may interact with the JAK/STAT signaling pathway and could possibly participate in the development of neoplastic essential thrombocythemia in female with recurrent immune thrombocytopenia.

## Figures and Tables

**Table 1:** MPN Ph(−) and ITP case reports.

Case number	1	2	3
Authors	Huang CE et al. [9]	Farhat et al. [10]	Sobas et al. [this report]

Sex	Female	Female	Female

ITP diagnosis	January 2009 (14 y.o.)	October 2001	July 1992 (45 y.o.)

ITP treatment	(1) DXM × 5 (2) Splenectomy (Aug. 2012)	(1) DXM × 4 (2) Igs (no data about doses)	(1) PD 1 mg/kg × 3 (1st and 2nd relapse) (2) After ET dgn: (i) 3rd ITP relapse: Igs 0,4 mg/kg × 5 days + AZT (ii) 4th ITP relapse: PD + AZT

ET diagnosis	August 2012: highly probable	March 2001	January 2013

JAK2 mutation	Positive: 11% allele burden	Positive	Positive

BM biopsy	Not done (patient refused)	Compatible with ET (2001)	Compatible with ET (2013)

MPN – ITP latency	Not known	ITP diagnosed 8 months after ET diagnosis (in 1994 patient was diagnosed with TTP)	ET diagnosed after 21 years of follow-up

Splenomegaly	No	No	No

ET treatment	No data	(1) Aspirin and ANA for 2 months, no response (2) HU	HU

Bone marrow (BM) biopsy, dexamethasone (DXM) 40 mg/day × 4 days, immunoglobulins (Igs), TTP (thrombotic thrombocytopenic purpura), ANA (anagrelide), HU (hydroxyurea), prednisone (PD), and azathioprine (AZT).
